# Burden of hydrocoele assessed from medical and surgical records in a lymphatic filariasis endemic country, Samoa

**DOI:** 10.1186/s41182-019-0179-0

**Published:** 2019-11-06

**Authors:** Tile A. Ah Leong-Lui, Patricia M. Graves, Take Naseri

**Affiliations:** 1 Ah Leong Clinic, Apia, Samoa; 2 James Cook University, Cairns, Australia; 3 Ministry of Health, Apia, Samoa

**Keywords:** Lymphatic filariasis, Hydrocoele, Samoa, Health information systems, Surgery

## Abstract

**Background:**

Samoa is a Pacific Island country that has long been known to have a high burden of lymphatic filariasis. Little has been documented about the burden of disability due to the chronic complications of the disease. We examined the rates of hydrocoele amongst the Samoan male population to better understand the situation.

**Methods:**

Information on numbers of suspected hydrocoele cases in men aged 18 years and older from 2006 to 2013 was sought using ICD-10 codes and/or keywords from three sources: the hospital patient information system plus the surgical clinic and operating theater records in Tupua Tamasese Meaole and Malietoa Tanumafili II hospitals in Samoa. Chart review of suspected hydrocoele cases was used to confirm the diagnosis of hydrocoele amongst suspected cases. The following data items were extracted from patient records where available: date of diagnosis, age, village, hydrocoele characteristics (duration, size, and volume), history and cause of injuries, whether lymphatic filariasis was a differential diagnosis, whether ultrasound scan was used to verify diagnosis, and details of any surgery performed. Population data were obtained from the Samoa Bureau of Statistics.

**Results:**

There were 535 suspected cases identified from the 3 sources between 2006 and 2013, of which 328 were diagnosed as hydrocoele; charts for 56 suspected cases (10.5%) could not be located. The mean age of men with hydrocoele was 49.2 years. The proportion of men aged ≥ 18 years diagnosed with hydrocoele over the study period was 0.62% (328/52,944). North West Upolu had the highest proportion amongst the four regions of Samoa (*p* < 0.001). The proportion of men presenting with hydrocoele increased with age (*p* < 0.001). 14.3% of patients had an injury that could have contributed to the hydrocoele. Only 4.0% of all patient records had lymphatic filariasis recorded as a differential diagnosis. 60.7% of all patients with hydrocoele had some form of surgery, with no difference between regions (*p* = 0.276). The majority of surgeries were hydrocoelectomies, where the tunica vaginalis is everted. The mean age of patients that had surgery was 48.2 years. It was difficult to estimate hydrocoele size and duration due to non-standardized way of reporting.

**Conclusions:**

This study used multiple sources to document the number of hydrocoele cases that presented annually to medical facilities in Samoa. This represents a minimum estimate of the burden since some cases may have not presented for treatment. The numbers presenting have fluctuated over the years (2006 to 2013), and improvements in the reporting system are needed. The health system needs to consider ways to address a large number of patients that still require surgery, as well as conducting follow-up of those that did receive surgery. Additionally, clinicians should consider lymphatic filariasis as a differential diagnosis for hydrocoeles.

## Background

Lymphatic filariasis (LF) is a disease caused by nematode worms transmitted by mosquitoes. The majority of infections (90%) are caused by *Wuchereria bancrofti* [1], which is the only species found in Samoa [2]. Worms inhabit the human lymph system and cause chronic inflammation and damage to the lymphatic vessels. This can lead to irreversible swelling of the scrotum in men, a complication known as hydrocoele. This develops slowly over many years and is lifelong unless alleviated by surgery.

Samoa is a Pacific Island nation with a tropical climate all year round [3], and the economy is highly dependent on tourism [4]. The population in 2011 was 187,820 with a landmass of 2785 km^2^ [5]. The population growth rate between 2006 and 2011 was 0.63% [5]. Samoa consists of 2 main islands, Upolu and Savaii. Apia on Upolu Island is the capital of Samoa. Statistically, the country is divided into 4 regions: Apia Urban Area (AUA), North West Upolu (NWU), Rest of Upolu (ROU), and Savaii (SAV). In 2016, the populations for the regions were 36,735, 62,390, 44,293, and 44,402, respectively. There are 8 established district hospitals, 2 newer district hospitals, and 2 subcenters in Samoa. There are also private clinics, which are mostly situated in Apia.

LF is one of the oldest diseases known to humans and Samoans alike [6], with accounts of chronic morbidity in the Samoan people reported as far back as 1882, and one which the government of Samoa has been trying to control as a public health problem for many years [2]. Mosquitoes implicated in the transmission of LF in Samoa include the highly efficient day- and night-biting vectors *Aedes polynesiensis* and *Aedes samoanus*, which are abundant in the country [2, 7]. Approximately 53% of houses are of open construction [5], making the population of Samoa vulnerable to mosquito-borne diseases.

In a renewed effort to control the disease, Samoa joined PacELF, the Pacific arm of the Global Programme to Eliminate Lymphatic Filariasis (GPELF), in 1999. The 2 pillars of the GPELF are to reduce disease transmission through mass drug administration of a combination of drugs and to alleviate suffering and disability from the chronic manifestations of the disease, namely lymphoedema of the limbs and hydrocoele [1, 2]. LF falls in the category of neglected tropical diseases, which often affect poor populations causing disability, stigmatization, and loss of work, thereby continuing the cycle of poverty for the victims and families [8]. To better address the latter goal of GPELF in endemic countries, it is important to understand the burden of complications.

In Samoa, little research has been done to understand the magnitude of the disease burden or its effects on society. However, it was known at the outset that hydrocoele surgery is one of the commonest surgeries performed in Samoa. With this in mind, we wanted to estimate the proportion of men with hydrocoele by region, age, and year as well as estimate the proportion of cases that had surgical treatment during the study period. Hydrocoele prevalence could be estimated by representative population surveys, but this would need large resources. Thus, we decided to first utilize existing routinely collected health system data to address the research questions. Hydrocoele takes many years to develop, and those affected may delay presentation for treatment, so the numbers of cases presenting to health facilities by year do not necessarily represent a true incidence. Nevertheless, the proportion of men presenting over the 8-year period provides a first estimate of the burden in the population.

## Results

From January 1, 2006, to December 31, 2013, we identified 535 patients aged ≥ 18 years as suspected hydrocoele cases who were eligible for further chart review. 10.5% of patient records could not be located (*N* = 56). 89.5% of patient files were found and reviewed (*N* = 479). Of these, 68.5% of patients met the case definition for hydrocoele (*N* = 328). Table 1 provides the summary of data completeness for the various data sources.

**Table 1:** Numbers of hydrocoele cases identified from various sources of data over the period 2006 to 2013

Information source	Eligible	Met case definition	Files not found
PATIS	502	314	42
Surgical clinic	23	11	8
Operating theater	10	3	6
Total	535	328	56

**Fig. 1:**
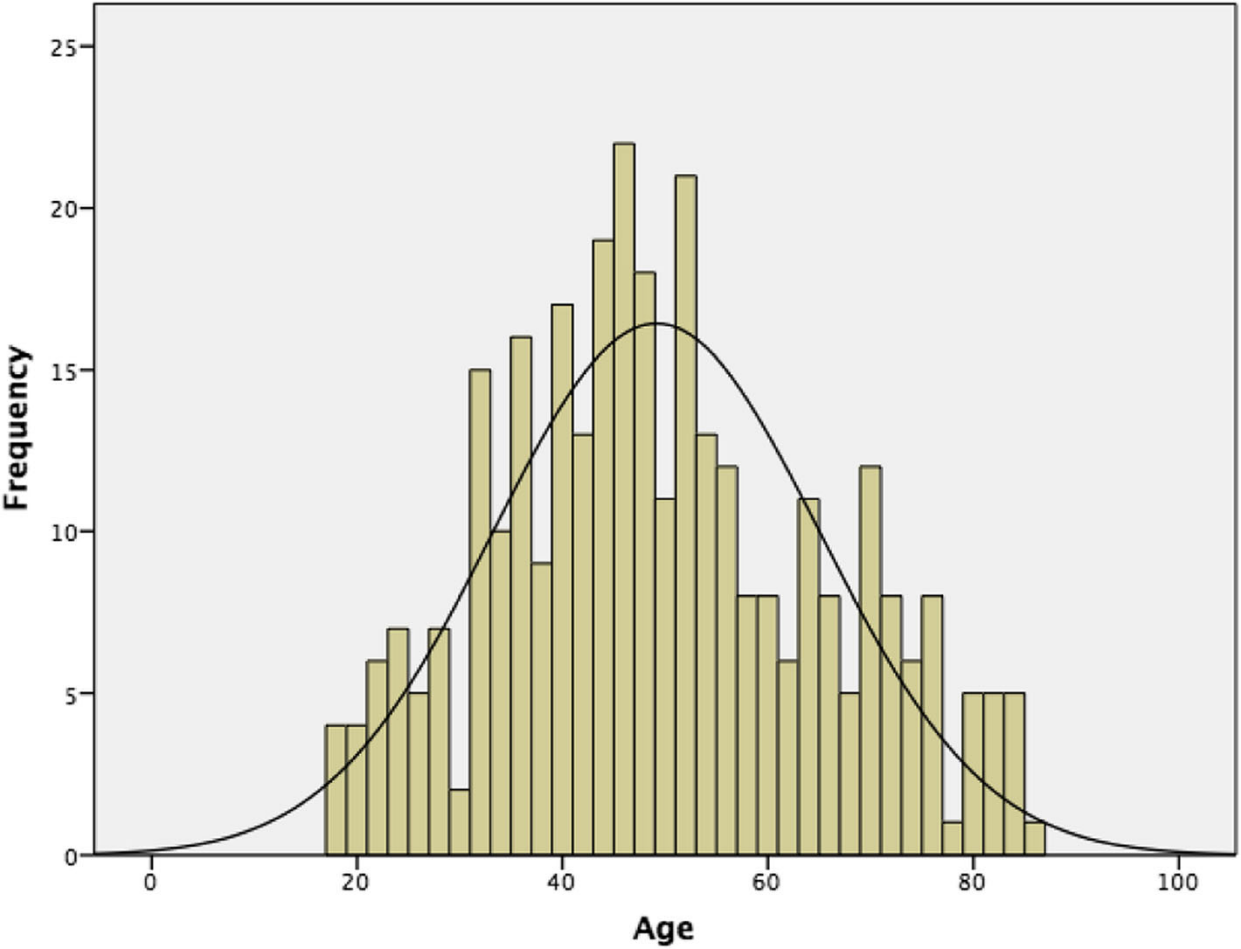
Age distribution of males over 18 years with hydrocoeles in Samoa

Figure 1 shows the age distribution of all males presenting with disease that fulfilled the case definition of hydrocoele. The mean age of patients with hydrocoele was 49.2 years, with a standard deviation of 15.9 years. The minimum age was 18 years old, and the maximum age was 85 years old.

### Rates of hydrocoele

The proportion of males ≥ 18 years old presenting with a hydrocoele over the 8 years of the study was 0.62% (95% CI 0.58–0.66%; 328 cases/52,944 population of men ≥ 18 years old (Census 2011)). The proportion of men with hydrocoele by region is depicted in Table 2, using population estimates from the 2011 census. The difference in proportions between regions was statistically significant (*p* < 0.001). The proportion was higher in NWU (0.81%) compared to the rest of the country combined (0.51%; *p* < 0.001).

**Table 2:** Proportion of men > 18 years with hydrocoele cases by regions

Region	Proportion
AUA	0.54 (57/10511)
NWU	0.81 (144/17689)
ROU	0.49 (62/12668)
SAV	0.50 (60/12076)

Unspecified location = 5 cases

Table 3 shows the proportion of men presenting with hydrocoele by year. A comparison of the proportions indicates a significant difference by year (*p* < 0.001). There was a significant increasing trend (*p* = 0.002), but this deviated from a linear trend (*p* = 0.013).

**Table 3:** Proportion of men ≥ 18 years with hydrocoele by year

Year	Cases	Total men > 18 years	Proportion
2006	23	50,876	0.05
2007	48	51,283	0.09
2008	32	51,693	0.06
2009	27	52,107	0.05
2010	38	52,524	0.07
2011	48	52,944	0.09
2012	59	53,368	0.11
2013	53	53,794	0.10

**Fig. 2:**
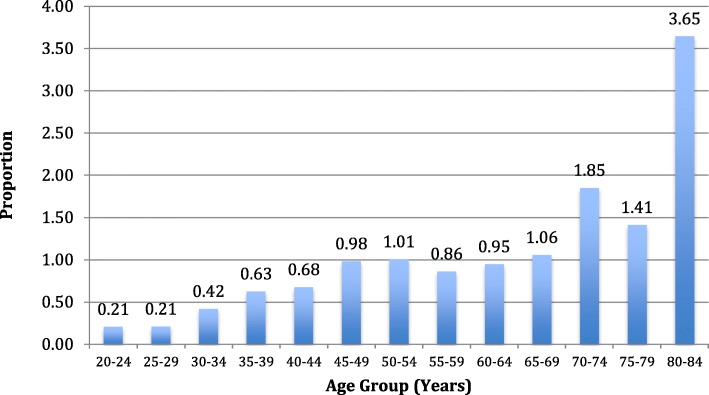
Proportion of men ≥ 18 years presenting with hydrocoele by age

Figure 2 shows the proportions of men presenting with hydrocoele by age group, showing a significant difference between age categories (*p* < 0.001) and a significant increasing trend with age (*p* < < 0.001) which was however nonlinear (*p* = 0.007).

### Surgery status

**Fig. 3:**
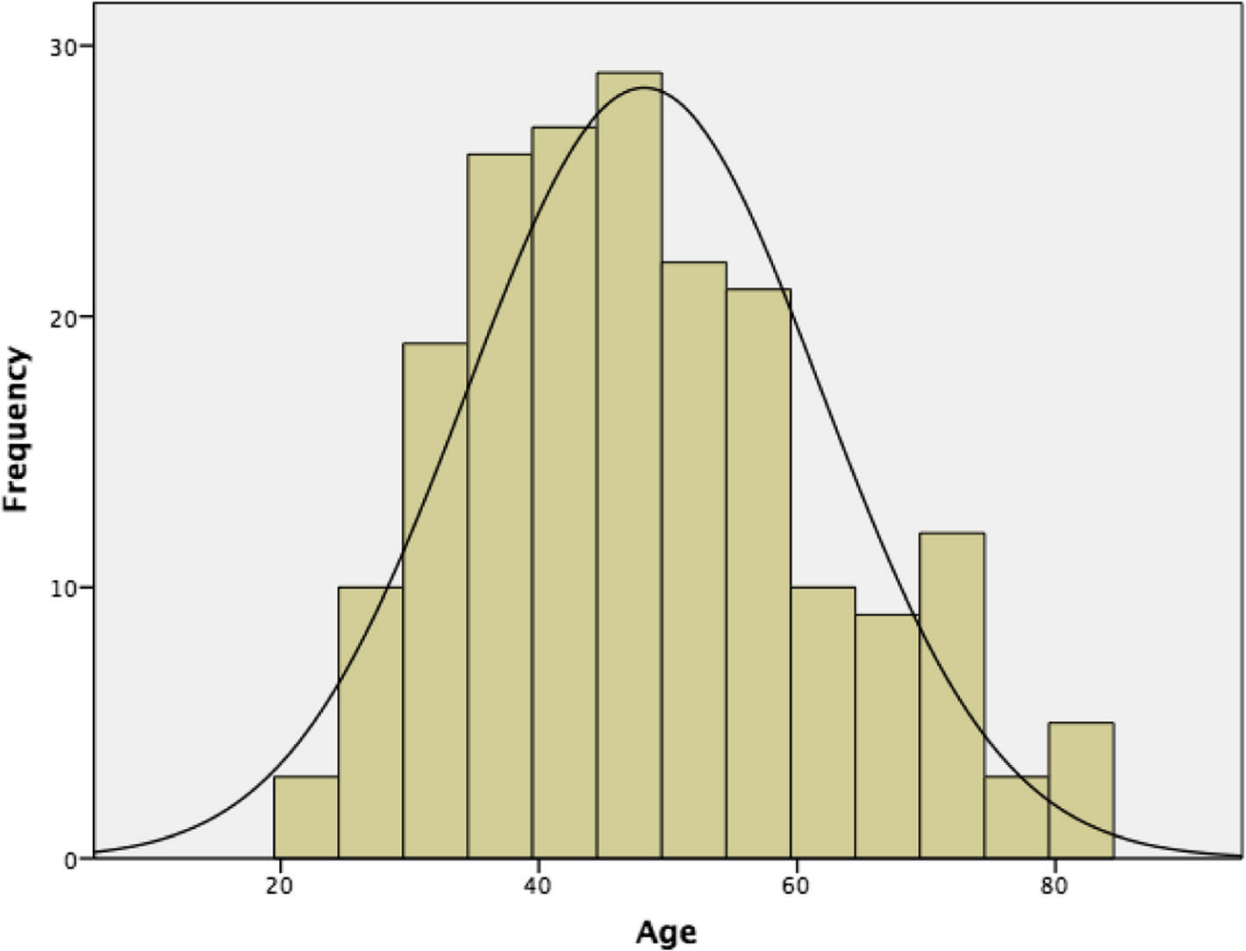
Age distribution of men > 18 years with hydrocoele that underwent surgery

60.7% of all patients with hydrocoeles over the study period underwent some form of surgery (*N* = 199). The mean age of patients that underwent surgery was 48.2 years with a standard deviation of 13.7 years. The minimum age was 22 years old, and maximum was 84 years old. Figure 3 illustrates the age distribution of males that underwent surgery.

Table 4 lists those that had surgery by region with 52.6%, 63.2%, 56.5%, and 68.3% for AUA, NWU, ROU, and SAV regions, respectively. There was no difference between regions in the proportion of cases that received surgery (*p* = 0.276).

**Table 4:** Surgery status of men with hydroceole by region

Region	Had surgery	No surgery	Total
AUA	30	27	57
NWU	91	53	144
ROU	35	27	62
Savaii	41	19	60
Unspecified location	2	3	5
Total	199	129	328

The majority of patients who had hydrocoelectomies (*N* = 169) had eversion of the tunica vaginalis (*N* = 159). If there were no surgical notes documented and sighted for a case, this was excluded from the analysis (*N* = 30). Other patients received procedures such as drainage of fluid or hernia repair to address concurrent hernia.

### Size, volume, and duration of hydrocoele

Hydrocoele sizes were difficult to analyze due to non-standardized ways of reporting. Terms such as grossly enlarged, gross swelling, massive, large, small, equal to a woman’s fist, or actual dimensions were used to describe the size. More importantly, the size was not recorded for many patients (*N* = 291).

For hydrocoele duration, 118 patients reported having had hydrocoeles for 0–4 years, 36 reported 5–9 years, and 35 reported having had hydrocoele for over 10 years. Many more (*N* = 120) had no documentation of hydrocoele duration, and the remainder were difficult to analyze due to the use of non-standardized terms such as many years, long time, and chronic (*N* = 19).

### Injury status and mechanism of injury

14.3% of all cases reported a history of trauma that could have contributed to the hydrocoele (*N* = 47). Of the 47 patients with a history of injury, 21 had a sports-related injury, namely cricket and rugby; 4 claimed to have been associated with heavy weight lifting; 4 had a history of fall; 8 were unspecified; and 10 were reported to be through other mechanisms such as carpentry work or kick in the groin by either a human or a horse. The majority did not have any documented history of injuries.

### Lymphatic filariasis as a differential diagnosis

Only 4.0% of patients (*N* = 13) with a hydrocoele over the study period were given a differential diagnosis of lymphatic filariasis (*N* = 328 patients).

### Ultrasound scan confirmation

16.9% of patients with a hydrocoele (*N* = 55) had an ultrasound confirmation of the diagnosis (*N* = 326).

## Discussion

Chronic long-term complications of LF have been a problem in the Samoan population since before the 1900s [6]. Similarly as described by Heffinger, other island nations suffered the same plight in the same period [6]. In 1962, the Government of Samoa started mass drug administration (MDA) with the goal of reducing the burden of complications due to LF amongst its people [2]. Five consecutive rounds of MDAs with either diethylcarbamazine (DEC) or DEC plus ivermectin were done in the 1990s [2]. Subsequently, in 1999, Samoa joined the PacELF programme, which redefined its control strategy for the disease to use DEC and albendazole for at least five rounds with more than 65% coverage, with the goal of interrupting transmission and eventually eliminating LF from the country [2]. Since then, 11 interrupted rounds of MDAs targeting the entire country with varied coverage rates have been implemented, with the last round in 2011 [2, 9].

Despite the many rounds of MDA with a combination of DEC and albendazole [2], LF remains endemic in the country [9]. In 2013, through a transmission assessment survey, it was found that LF was still endemic in NWU with a high prevalence of antigenemia in young children in comparison to other regions of Samoa [9]. This led to the decision by the Ministry of Health to continue to implement two more rounds of MDA in this region [9]. Similarly, NWU is well documented as a problem region for LF in previous research done by Dr. Ichimori and colleagues [2].

Even after transmission of LF has been interrupted, people with the chronic complications remain for many years. Hydrocoele is described as the commonest complication of LF in males and is much more common than lymphoedema [10]. Despite this, no available data exist on the burden of hydrocoele in Samoa. Thus, this research estimated the proportion of patients that received treatment and provided insight into the epidemiology of hydrocoele in the Samoan population.

Surgery, in particular, the subtotal excision of the tunica vaginalis (the outermost layer of the scrotum), was the recommended treatment for hydrocoeles by the World Health Organization (WHO) in an unofficial meeting in 2002 [10, 11]. Updated guidelines for surgery were discussed at an informal consultation at WHO in 2019 [12], and a systematic review has summarized the different techniques performed [13]. Hydrocoele surgery is performed as a day case in most hospitals, which means that in many settings, this is done as an elective procedure [10]. Consequently, surgeries are not prioritized, and many males in Samoa are not given the definitive treatment which they desire.

This study was restricted to men ≥ 18 years old, as we did not want to include anyone with a congenital hydrocoele. Often, children with benign congenital problems receive surgeries when they are in older childhood.

As this study shows, the proportion of men ≥ 18 years old presenting with hydrocoele was 0.62%. The proportion increased with age. A significantly higher proportion of men presented in NWU region (0.81%) than the other regions. This region as mentioned earlier failed a transmission assessment survey in 2013 [9], and transmission is still ongoing.

More than half of all patients underwent some form of surgery for their hydrocoele, and the majority of surgeries performed were hydrocoelectomies with eversion of the tunica vaginalis which is not in line with WHO’s recommendation of subtotal excision of the tunica vaginalis [10, 11]. The proportion of men with hydrocoeles that received surgery was not significantly different by region.

There was no evidence of a decreasing trend in the presentation of hydrocoeles by year: in fact, the trend was in the opposite direction. This is a worrying trend suggesting that more effort is required to bring LF transmission under control in Samoa. However, we cannot tell whether this is due to the increasing incidence or to increased presentation of longstanding cases.

In relation to LF as a contributing factor to hydrocoele, it is quite interesting to know that only 4% of patients with hydrocoeles had LF as a differential diagnosis for the cause of their problem. In LF-endemic countries, WHO reports that all hydrocoeles are considered due to LF until proven otherwise [10]. One of the reasons that could explain this low proportion is the lack of clinician awareness and knowledge of this ancient disease.

It is frequently stated in Samoa that hydrocoele is associated with Samoan cricket. An injury to the groin or directly to the scrotum by the hard, solid cricket ball is thought to cause hydrocoele. However, in this study, only 47 patient records had a documentation of an injury, and fewer than half of these were associated with sports, namely Samoan cricket and rugby.

Hydrocoele duration and size were difficult to estimate, as there is no standardized way in which these are recorded clinically. As Capuano and Capuano have suggested, it is important for LF-endemic countries to have a standard way of reporting for international comparison, but most importantly, to determine the best surgical procedure for patients [11]. We were not able to detect an association between hydrocoele volume and duration, since standardized information on volume was lacking and numbers were too small for this purpose.

## Conclusions

This study used multiple sources to document the number of hydrocoele cases that presented annually to medical facilities in Samoa between 2006 and 2013. The overall proportion of men presenting with hydrocoeles in Samoa was 0.62%, with North West Upolu region having the highest burden. The number of cases identified over this time period (328) represents a minimum estimate of the burden, since some cases may not have presented to health facilities. The numbers reported have fluctuated over the years (2006 to 2013), and improvements in the reporting system are needed. The health system needs to consider ways to address a large number of patients that still require surgery, as well as conducting follow-up of those that did receive surgery, which was 60.7% of all hydrocoele cases. Because LF is an ancient disease, clinicians should be reminded and made aware that hydrocoele is a complication of the disease and should be considered a differential diagnosis for hydrocoeles. Surgeons should also review how they classify the severity of hydrocoele for standardization and best treatment option for patients.

## Methodology

### Study location and setting

Chart review was performed for all eligible participants from January 1, 2006, to December 31, 2013, at Tupua Tamasese Meaole (TTM) Hospital on Upolu Island and Malietoa Tanumafili II (MT II) Hospital on Savaii Island. These hospitals were selected as they are referral hospitals for Upolu and Savaii, respectively. Major surgeries, e.g., hydrocoelectomies, are only performed there. Any patient needing a surgical review is referred to these hospitals regardless of the rural health facility at which they present.

**Fig. 4:**
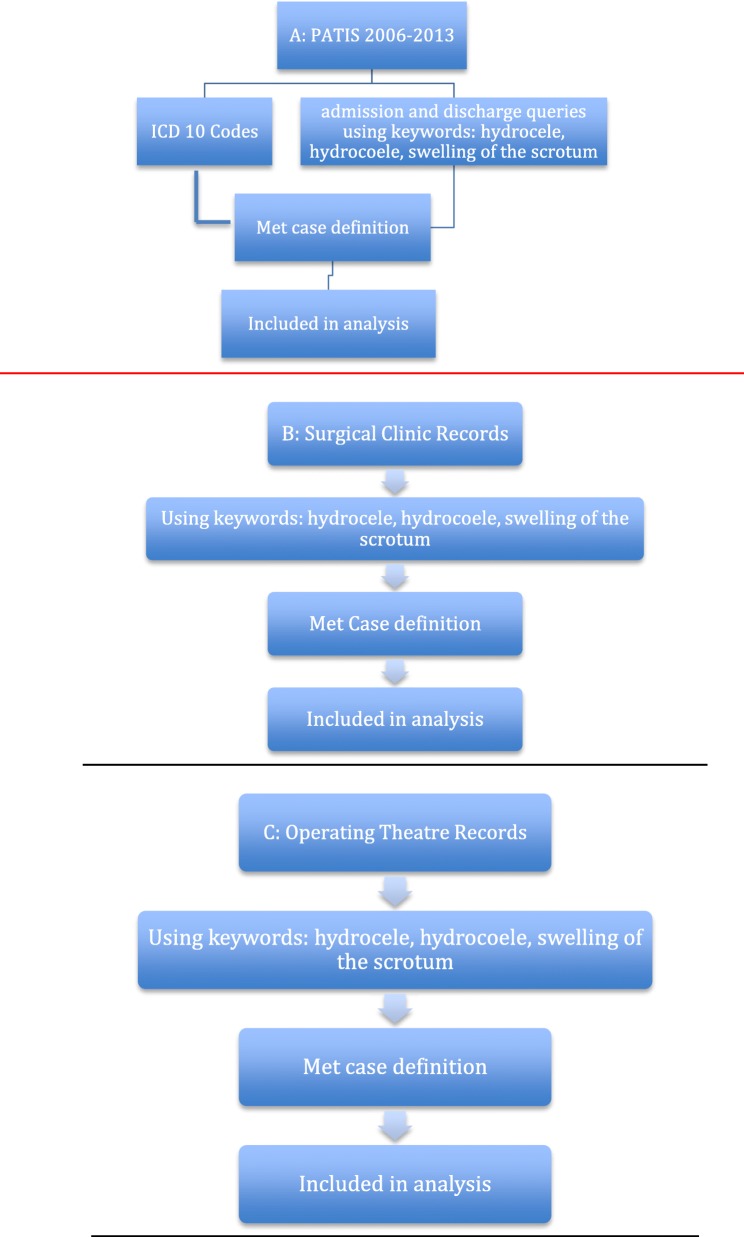
Study methodology and sources of information on cases. **A** PATIS information system. **B** Surgical clinic records. **C** Operating theater records

The patient information system (PATIS) was set up in 2006 at these two hospitals, and the study used data from that year until 2013. The PATIS system uses ICD-10 codes to classify patients. In addition to PATIS, a review of surgical clinic and operating theater records was done to ensure the maximum number of patients was captured (Fig. 4).

### Inclusion criteria

Males ≥ 18 years old, with a diagnosis of “hydrocele” or “hydrocoele” or “swelling” of the scrotum or scrotal region from January 1, 2006, to December 31, 2013.

### Exclusion criteria

All females and males ≤ 17 years old and anyone who did not have a diagnosis of “hydrocele” or “hydrocoele” or “swelling” of the scrotum or scrotal from January 1, 2006, to December 31, 2013. Males ≤ 17 years old were excluded as we did not want to include anyone with a congenital hydrocoele that had surgery in late childhood.

### Case definition for hydrocoele

To ensure that patients indeed had hydrocoeles, diagnoses of hydrocoele had to be clearly documented on the date of visit or 7 days post-visit date. Otherwise, for patients that presented multiple times, any diagnosis of hydrocoele had to be noted on any given date. Additionally, if the diagnosis was amended during surgery to alternative diagnoses, this was not counted as a case or excluded. The reason for the lapse in clinic date is because data entry does not always happen on the same day; rather, it could be entered a few days later when PATIS is interrupted.

### PATIS (Fig. 4A)

To generate the list of patients with potential hydrocoeles, International Classification system for Diseases (ICD) 10 codes were used to run queries which included the following:

- N43 (hydrocoele and spermatocele)
- N43.0 (encysted hydrocoele)
- N43.1 (infected hydrocoele)
- N43.2 (other hydrocoele)
- N43.3 (hydrocoele, unspecified)

This was done as we found during preliminary preparations that patients with hydrocoele were coded differently using the above codes.

To ensure that all potential patients with hydrocoeles were captured in the study, admission and discharge queries were also run using the keywords “hydrocele,” “hydrocoele,” and “swelling” (of the scrotum or scrotal) for both hospitals. Clinical staff used the two spellings interchangeably, so we needed to use both to capture all cases. The main reason why this additional step was needed was because outputs from the previous method only included primary and secondary diagnoses. Third or fourth diagnoses of hydrocoeles were not included in the first method. It was also critical to pull out files for “scrotal swelling” to confirm a diagnosis of hydrocoele.

### Surgical clinic records (Fig. 4B)

These records were sighted to ensure that patients whose medical records were not entered into PATIS were included in the study. The key terms “hydrocele,” “hydrocoele,” or “swelling” (of the scrotum or scrotal) were used to identify potential study participants.

### Operating theater records (Fig. 4C)

These records were sighted for two purposes: firstly, to determine the proportion of all patients with hydrocoeles that had undergone surgery, and secondly, to ensure that patients not captured in PATIS and surgical clinic records (but which were recorded in the operating theater records) were included in the study. The key terms “hydrocele” or “hydrocoele” or “swelling” (of the scrotum or scrotal) were identified to determine eligibility.

All three sources were needed to give a better estimate of the proportion of men presenting with hydrocoele in the Samoan population. Patient unique identifier numbers or national health numbers (NHN) were used to remove duplicates and ensure that single entities remained in the final list of eligible participants.

### Statistical analysis

The distribution and proportion of hydrocoele cases by age and region and cases that had surgery were analyzed using IBM SPSS version 22 and/or STATA 14. Statistical tests used were Pearson’s chi-square and chi-square test for trend.

Denominators for estimating proportions of men presenting with hydrocoeles were obtained from the Census. Theoretically, men with hydrocoeles or who had had surgery (even prior to his study) should have been deducted from the denominator as they are not at risk. However, this proportion is very small compared to the overall number of men at risk.

## Study limitations

### Patient information system

A common problem with PATIS is that some patients have two unique identifier numbers. Coding may also be inaccurate. Duplicates were resolved using NHN numbers, but it is possible that we may have missed some patients that were coded incorrectly.

### Surgical clinic data

We were able to capture some patients with hydrocoele through analysis of surgical clinic data that were not recorded in PATIS. However, we found that some records were either missing or inadequately documented.

### Patient files

At the outset, we anticipated that some files would be difficult to locate. To mitigate this, a second list which contained the missing files from the first round was handed over to medical records to locate. After several attempts, some files were still unable to be located (*N* = 56).

### Surgical theater records

When clinical services moved between hospitals, theater books were misplaced in the process. Occasionally, it was very difficult to locate unique identifier numbers for several patients (and therefore their charts) but their records were captured in the operating theater and surgical clinic records.

### Study design

It is likely that some patients with hydrocoeles were not captured because they may not have presented at all or have presented to district hospitals and health centers which did not have PATIS. Examination of the surgical records mitigated the latter problem to some extent.

Limitations exist in the quality of information that was recorded in patient files. Brief documentation resulted in missing data items in some cases.

We also note that village of residence is not always accurate as patient demographics are not updated regularly, especially in PATIS. Consequently, where village or region is concerned, results should be interpreted with caution.

## Data Availability

The data is the property of the Government of Samoa and is not publically available.

## Abbreviations

AUA: Apia Urban Area
GPELF: Global Programme to Eliminate Lymphatic Filariasis
LF: Lymphatic filariasis
MT II: Malietoa Tanumafili II Hospital on Savaii Island
NWU: North West Upolu
PacELF: Pacific Programme to Eliminate Lymphatic Filariasis
PATIS: Patient information system
ROU: Rest of Upolu
SAV: Savaii
TTM: Tupua Tamasese Meaole Hospital on Upolu Island
WHO: World Health Organization
